# Metabolic engineering of *Escherichia coli* for production of (2*S*,3*S*)-butane-2,3-diol from glucose

**DOI:** 10.1186/s13068-015-0324-x

**Published:** 2015-09-15

**Authors:** Haipei Chu, Bo Xin, Peihai Liu, Yu Wang, Lixiang Li, Xiuxiu Liu, Xuan Zhang, Cuiqing Ma, Ping Xu, Chao Gao

**Affiliations:** State Key Laboratory of Microbial Technology, Shandong University, Jinan, 250100 People’s Republic of China; State Key Laboratory of Microbial Metabolism, School of Life Sciences and Biotechnology, Shanghai Jiao Tong University, Shanghai, 200240 People’s Republic of China; Rizhao Entry-Exit Inspection and Quarantine Bureau, Rizhao, 276800 People’s Republic of China

**Keywords:** (2*S*,3*S*)-Butane-2,3-diol, Metabolic engineering, α-Acetolactate synthase, *meso*-Butane-2,3-diol dehydrogenase

## Abstract

**Background:**

Butane-2,3-diol (2,3-BD) is a fuel and platform biochemical with various industrial applications. 2,3-BD exists in three stereoisomeric forms: (2*R*,3*R*)-2,3-BD, *meso*-2,3-BD and (2*S*,3*S*)-2,3-BD. Microbial fermentative processes have been reported for (2*R*,3*R*)-2,3-BD and *meso*-2,3-BD production.

**Results:**

The production of (2*S*,3*S*)-2,3-BD from glucose was acquired by whole cells of recombinant *Escherichia coli* coexpressing the α-acetolactate synthase and *meso*-butane-2,3-diol dehydrogenase of *Enterobacter cloacae* subsp. *dissolvens* strain SDM. An optimal biocatalyst for (2*S*,3*S*)-2,3-BD production, *E. coli* BL21 (pETDuet–P_T7_–*budB*–P_T7_–*budC*), was constructed and the bioconversion conditions were optimized. With the addition of 10 mM FeCl_3_ in the bioconversion system, (2*S*,3*S*)-2,3-BD at a concentration of 2.2 g/L was obtained with a stereoisomeric purity of 95.0 % using the metabolically engineered strain from glucose.

**Conclusions:**

The engineered *E. coli* strain is the first one that can be used in the direct production of (2*S*,3*S*)-2,3-BD from glucose. The results demonstrated that the method developed here would be a promising process for efficient (2*S*,3*S*)-2,3-BD production.

**Electronic supplementary material:**

The online version of this article (doi:10.1186/s13068-015-0324-x) contains supplementary material, which is available to authorized users.

## Background

Butane-2,3-diol (2,3-BD) is a promising commodity biochemical that can be produced by biotechnological routes. It can be used as a starting material for the synthesis of bulk chemicals including methylethylketone, gamma-butyrolactone and 1,3-butadiene [1–3]. Additionally, the heating value of 2,3-BD (27,200 J/g) is comparable with that of other liquid fuels, e.g., methanol (22,100 J/g) and ethanol (29,100 J/g). Thus, 2,3-BD can also be used as a liquid fuel or fuel additive [1–3]. There are three isomeric forms of 2,3-BD: (2*R*,3*R*)-2,3-BD, *meso*-2,3-BD and (2*S*,3*S*)-2,3-BD. Optically pure 2,3-BD can act as an excellent building block in asymmetric synthesis of valuable chiral chemicals [1–3].

2,3-BD can be produced via chemical or biotechnological routes [1, 4–10]. The selective production of optically pure 2,3-BD through chemical processes is difficult to control, complicated and expensive to perform. Furthermore, the optical purity of 2,3-BD produced via chemical processes was rather low. Biotechnological routes have emerged as the preferred methods for the optically pure 2,3-BD production [1–3]. Although many microorganisms could be used to efficiently produce 2,3-BD, *Paenibacillus polymyxa* is the only native producer of optically pure (2*R*,3*R*)-2,3-BD [11–13]. Thus, specific strains of *Escherichia coli*, *Saccharomyces cerevisiae*, *Enterobacter cloacae* and *Bacillus licheniformis* have been engineered for optically pure 2,3-BD production in recent years [14–21]. For example, *S. cerevisiae* has been engineered as a heterologous host for (2*R*,3*R*)-2,3-BD production [16]. The highest concentration of (2*R*,3*R*)-2,3-BD was more than 100 g/L. 73.8 g/L of *meso*-2,3-BD can also be produced using systematic metabolic engineered *E. coli* BL21/pET-RABC from glucose [17]. However, no fermentative method for (2*S*,3*S*)-2,3-BD production has been reported.

Due to difficulties in the direct fermentative production of (2*S*,3*S*)-2,3-BD, biocatalysis has emerged as the only reported route for (2*S*,3*S*)-2,3-BD production [22–28]. Racemic acetoin, diacetyl or a mixture of three isomeric forms of 2,3-BD have been used as substrates for (2*S*,3*S*)-2,3-BD production [22–28]. The highest published concentration of (2*S*,3*S*)-2,3-BD produced from the mixture of 2,3-BD was 2.4 g/L, with a low yield of 0.12 g/g [28]. With pure diacetyl as substrate and formate for cofactor regeneration, 31.7 g/L (2*S*,3*S*)-2,3-BD was produced [23]. However, all of the above substrates were rather expensive. Thus, production of (2*S*,3*S*)-2,3-BD with high optical purity from cheap substrates, such as glucose, is rather desirable.

Until now, a microorganism that could directly produce (2*S*,3*S*)-2,3-BD from glucose through bioconversion has never been reported. In this work, a recombinant *E. coli* was constructed through coexpressing the α-acetolactate synthase (ALS) and *meso*-butane-2,3-diol dehydrogenase (*meso*-BDH) of *E. cloacae* subsp. *dissolvens* strain SDM. Then, (2*S*,3*S*)-2,3-BD was produced from glucose using whole cells of the recombinant *E. coli*. The process presented in this work could be a promising alternative for the biotechnological production of optically pure (2*S*,3*S*)-2,3-BD.

## Results and discussion

### Design of the metabolic pathway for (2*S*,3*S*)-2,3-BD from glucose

Many bacteria such as *Klebsiella pneumoniae*, *Klebsiella oxytoca* and *E. cloacae* can ferment sugars to a mixture of (2*R*,3*R*)-2,3-BD, *meso*-2,3-BD and (2*S*,3*S*)-2,3-BD [1, 5, 29–32]. In previous studies, the mechanism of 2,3-BD stereoisomer formation in *K. pneumoniae* was identified [33, 34]. In the 2,3-BD-producing process, carbohydrate must first be converted to pyruvate. Then, three key enzymes, i.e., ALS, α-acetolactate decarboxylase (ALDC) and butane-2,3-diol dehydrogenase (BDH), are involved in 2,3-BD production from pyruvate. Two molecules of pyruvate are condensed to α-acetolactate by ALS. Then, ALDC catalyzes the decarboxylation of α-acetolactate to produce (3*R*)-acetoin. (3*R*)-acetoin will be reduced to *meso*-2,3-BD and (2*R*,3*R*)-2,3-BD by *meso*-BDH and (2*R*,3*R*)-BDH, respectively. α-Acetolactate is unstable and can also be catalyzed through nonenzymatic oxidative decarboxylation to produce diacetyl. (2*S*,3*S*)-2,3-BD, the target product of this study, could only be produced by the *meso*-BDH-catalyzed two-step reduction of diacetyl [33, 34]. Due to the low concentration of diacetyl in microbial fermentation, (2*S*,3*S*)-2,3-BD can only be produced at low concentration and stereoisomeric purity.

**Fig. 1:**
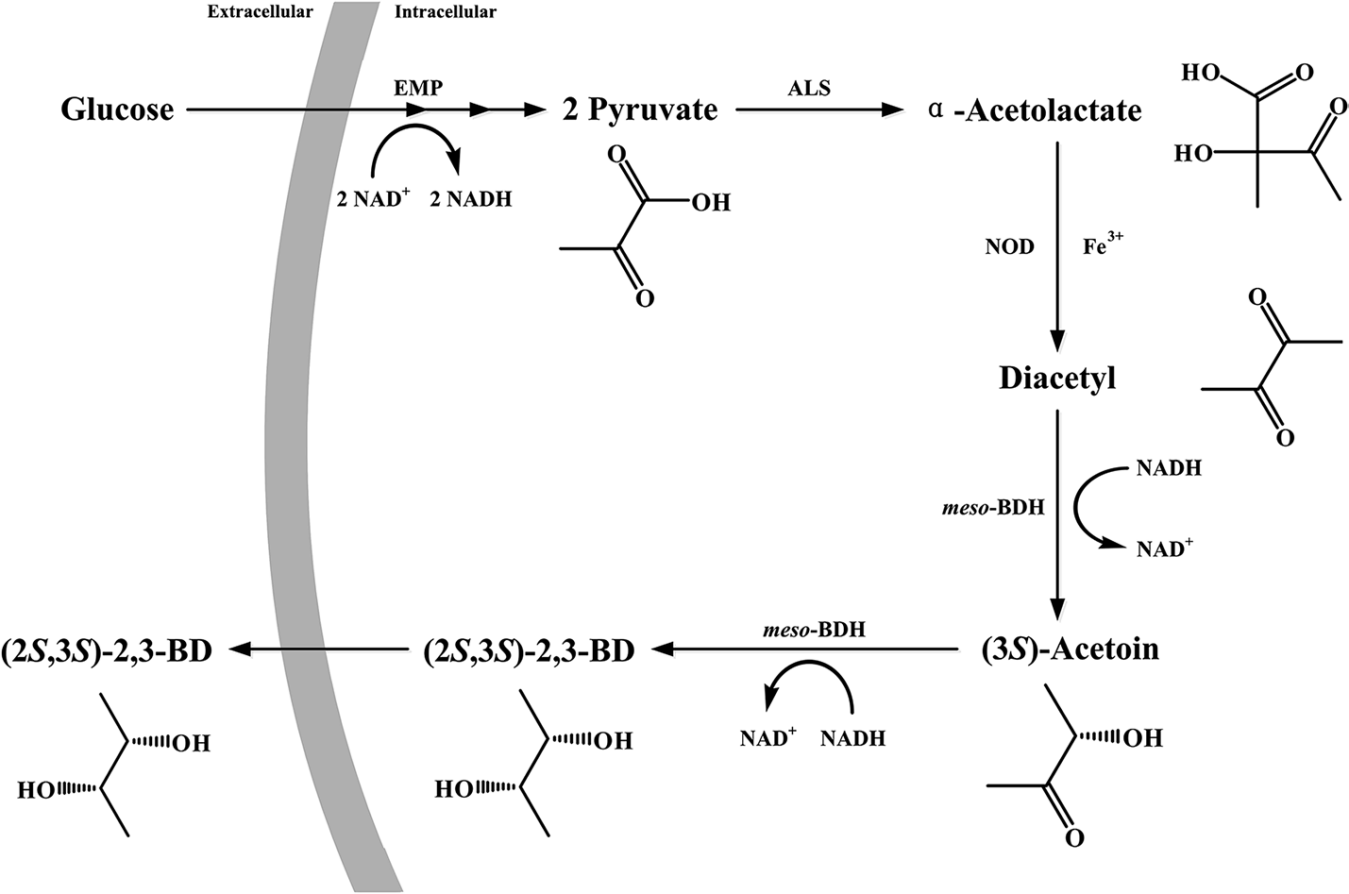
Technology road map for (2*S*, 3*S*)-2,3-BD production from glucose by recombinant *E. coli*. *ALS* α-acetolactate synthase, *meso*-*BDH* *meso*-butane-2,3-diol dehydrogenase, *NOD* non-enzymatic oxidative decarboxylation, *EMP* Embden–Meyerhof–Parnas pathway

Based on the native (2*S*,3*S*)-2,3-BD producing process mentioned above, we designed a metabolic pathway for (2*S*,3*S*)-2,3-BD production from glucose by recombinant *E. coli*. Pyruvate is firstly formed from glucose via the endogenous Embden–Meyerhof–Parnas pathway (glycolysis) in *E. coli*. Then, α-acetolactate is produced from pyruvate by the exogenously expressed ALS. Diacetyl can be produced from α-acetolactate by nonenzymatic oxidative decarboxylation. Finally, diacetyl would be reduced to (3*S*)-acetoin and then to (2*S*,3*S*)-2,3-BD by the exogenously expressed *meso*-BDH in the recombinant *E. coli* (Fig. 1).

### Construction of recombinant plasmids for (2*S*,3*S*)-2,3-BD

In a previous report, the genes related to 2,3-BD production in different strains were cloned into *E. coli* BL21(DE3) and the 2,3-BD synthesis abilities of the recombinant *E. coli* strains were assayed [17]. The recombinant *E. coli* expressing the genes from *E. cloacae* subsp. *dissolvens* SDM had the best ability to produce 2,3-BD. Thus, the ALS encoded by *budB* in *E. cloacae* subsp. *dissolvens* SDM was selected for the production of precursors of (2*S*,3*S*)-2,3-BD (α-acetolactate and diacetyl) in recombinant *E. coli*. Additionally, besides the reduction of (3*R*)-acetoin to produce *meso*-2,3-BD, the *meso*-BDH encoded by *budC* in strain SDM could also catalyze the two-step reduction of diacetyl to produce (2*S*,3*S*)-2,3-BD. The recombinant *E. coli* strains expressing the *meso*-BDH have been used in the efficient production of (2*S*,3*S*)-2,3-BD from diacetyl [23]. The highly active *meso*-BDH was also used in the (2*S*,3*S*)-2,3-BD production process.

**Fig. 2:**
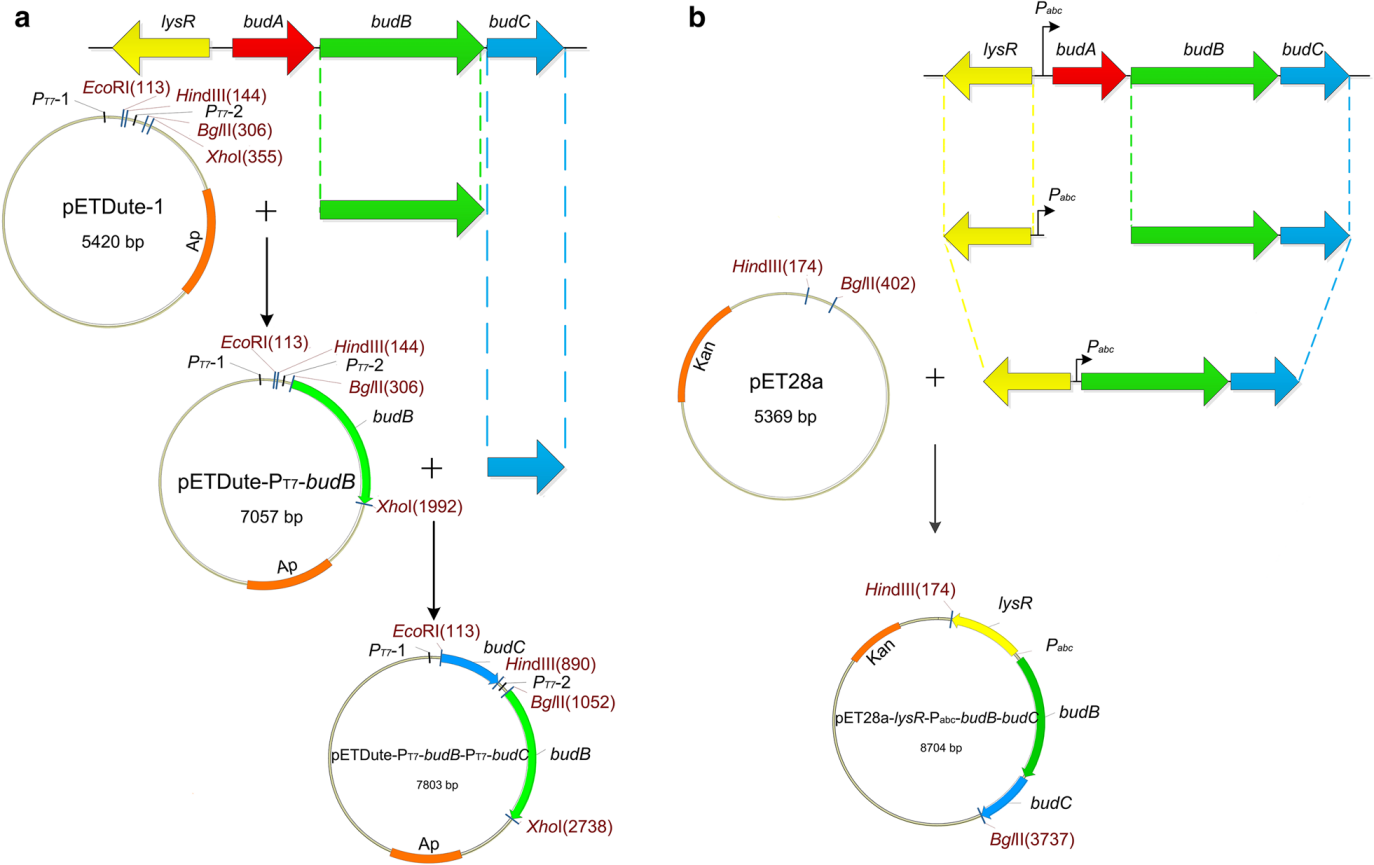
Construction of recombinant vectors for ALS and *meso*-BDH expression. **a** Construction of pETDuet–P_T7_–*budB*–P_T7_–*budC.* **b** Construction of pET28a–*lysR*–P_abc_–*budB*–*budC*. *lysR*, the gene encoding the transcriptional regulator; *budB*, the gene encoding ALS; *budA*, the gene encoding ALDC; *budC*, the gene encoding *meso*-BDH, *P*_*abc*_, the predicted promoter of the 2,3-BD pathway gene cluster from *E. cloacae* subsp. *dissolvens* strain SDM

As shown in Fig. 2, the genes encoding ALDC (accession number: 392323960), ALS (accession number: 392323961) and* meso*-BDH (accession number: 392323962) are sequentially clustered in one operon and under control of transcriptional regulation protein AlsR in *E. cloacae*. Two separate plasmids, pETDuet–P_T7_–*budB*–P_T7_–*budC* and pET28a–*lysR*–P_abc_–*budB*–*budC*, were used for expression of the *budB* and *budC* genes of *E. cloacae* subsp. *dissolvens* SDM in recombinant *E. coli* (Fig. 2). In a previous report, the 2,3-BD pathway genes of strain SDM were expressed under the control of different types of promoters. The recombinant *E. coli* strain with the native promoter (*P*_*abc*_) of 2,3-BD synthesis gene cluster of strain SDM had the best ability to produce 2,3-BD [17]. Thus, in pET28a–*lysR*–P_abc_–*budB*–*budC*, the genes *lysR*, *budB*, *budC* and *P*_*abc*_ of *E. cloacae* subsp. *dissolvens* SDM were ligated through gene splicing by overlap extension and cloned into the multiple clone site of pET28a. The expression of both *budB* and *budC* was also under the control of transcriptional regulation protein AlsR and the promoter *P*_*abc*_ of the 2,3-BD pathway gene cluster of strain SDM (Fig. 2b). In pETDuet–P_T7_–*budB*–P_T7_–*budC*, *budB* and *budC* were cloned into the two multiple clone sites of pETDuet-1 and under the control of the promoter *P*_*T7*_ (Fig. 2a).

The *budB* and *budC* genes were successfully cloned from *E. cloacae* subsp. *dissolvens* SDM and then inserted into pETDuet-1 to get pETDuet–P_T7_–*budB*–P_T7_–*budC* (Additional file 1: Figure S1). The fragment *budB*–*budC* amplified from the genomic DNA of SDM was about 2500 bp; while the fragment *lysR*–P_abc_ amplified from the genomic DNA of SDM was about 1000 bp (Additioanl file 1: Figure S1). These two fragments were ligated through recombinant PCR to get fragment *lysR*–P_abc_–*budB*–*budC*. This fragment was inserted into pET28a and resulted in plasmid pET28a–*lysR*–P_abc_–*budB*–*budC*.

### Production of (2*S*,3*S*)-2,3-BD by different recombinant *E. coli* strains

The constructed expression vectors were transformed into *E. coli* BL21(DE3) and the 2,3-BD synthesis abilities of the whole cells of recombinant strains were assayed. The 20 mL reaction mixtures were incubated at 30 °C and 180 rpm in a 50-mL flask.

The concentrations of glucose and whole cells of recombinant *E. coli* strains were 40 g/L and 5 g dry cell weight (DCW)/L, respectively. After 24 h bioconversion, the concentrations of 2,3-BD and glucose in the reaction mixture were determined. As shown in Table 1, *E. coli* BL21 (pETDuet-1) and BL21 (pET28a–*lysR*–P_abc_–*budB*–*budC*) could not produce (2*S*,3*S*)-2,3-BD within 24 h. *E. coli* BL21 (pETDuet–P_T7_–*budB*–P_T7_–*budC*) had a higher ability to produce 2,3-BD. 2,3-BD at a concentration of 1.14 g/L was produced by *E. coli* BL21 (pETDuet–P_T7_–*budB*–P_T7_–*budC*) within 24 h. The strain also showed the highest 2,3-BD yield among the strains harboring different recombinant plasmids (Table 1).

**Table 1:** Glucose consumption, product and yield analyses of *E. coli* strains harboring different vectors in 24 h flask cultures

Strain	Glucose consumed (g/L)	2,3-BD (g/L)	2,3-BD yield (g/g)
*E. coli* BL21 (pETDuet-1)	12.00 ± 0.00	ND	0.00
*E. coli* BL21 (pET28a–*lysR*–P_abc_–*budB*–*budC*)	14.00 ± 0.00	ND	0.00
*E. coli* BL21 (pETDuet–P_T7_–*budB*–P_T7_–*budC*)	13.67 ± 0.58	1.14 ± 0.01	0.08

Data are the mean ± standard deviations (SDs) from three parallel experiments

The activities of ALDC, ALS, and BDH in the recombinant strains were also assayed (Table 2). Consistent with the result of 2,3-BD production, *E. coli* BL21 (pETDuet-1) exhibited low ALS and BDH activities. *E. coli* BL21 (pETDuet–P_T7_–*budB*–P_T7_–*budC*) showed the highest ALS and *meso*-BDH activities. No ALDC activity could be detected in all of the *E. coli* strains. Since the concentration of 2,3-BD obtained and the ALS and *meso*-BDH activities of *E. coli* BL21 (pETDuet–P_T7_–*budB*–P_T7_–*budC*) were higher than that of other recombinant strains, *E. coli* BL21 (pETDuet–P_T7_–*budB*–P_T7_–*budC*) was chosen for further investigation.

**Table 2:** Enzyme activities of *E. coli* strains harboring different vectors in 24 h flask cultures

Strain	ALS activity (U/mg)	ALDC activity (U/mg)	BDH activity (U/mg)		
			Reduction		Oxidation
			Diacetyl	Acetoin	*meso*-2,3-BD
*E. coli* BL21 (pETDuet-1)	0.03 ± 0.04	ND	0.18 ± 0.01	0.20 ± 0.04	ND
*E. coli* BL21 (pET28a–*lysR*–P_abc_–*budB*–*budC*)	0.16 ± 0.03	ND	1.79 ± 0.17	1.17 ± 0.09	0.38 ± 0.02
*E. coli* BL21 (pETDuet–P_T7_–*budB*–P_T7_–*budC*)	1.67 ± 0.06	ND	7.20 ± 1.21	4.20 ± 0.23	2.80 ± 0.20

Data are the mean ± standard deviations (SDs) from three parallel experiments

*ALS* α-acetolactate synthase, *ALDC* α-acetolactate decarboxylase, *BDH* butane-2,3-diol dehydrogenase

### Effect of pH on production of (2*S*,3*S*)-2,3-BD by recombinant *E. coli*

**Fig. 3:**
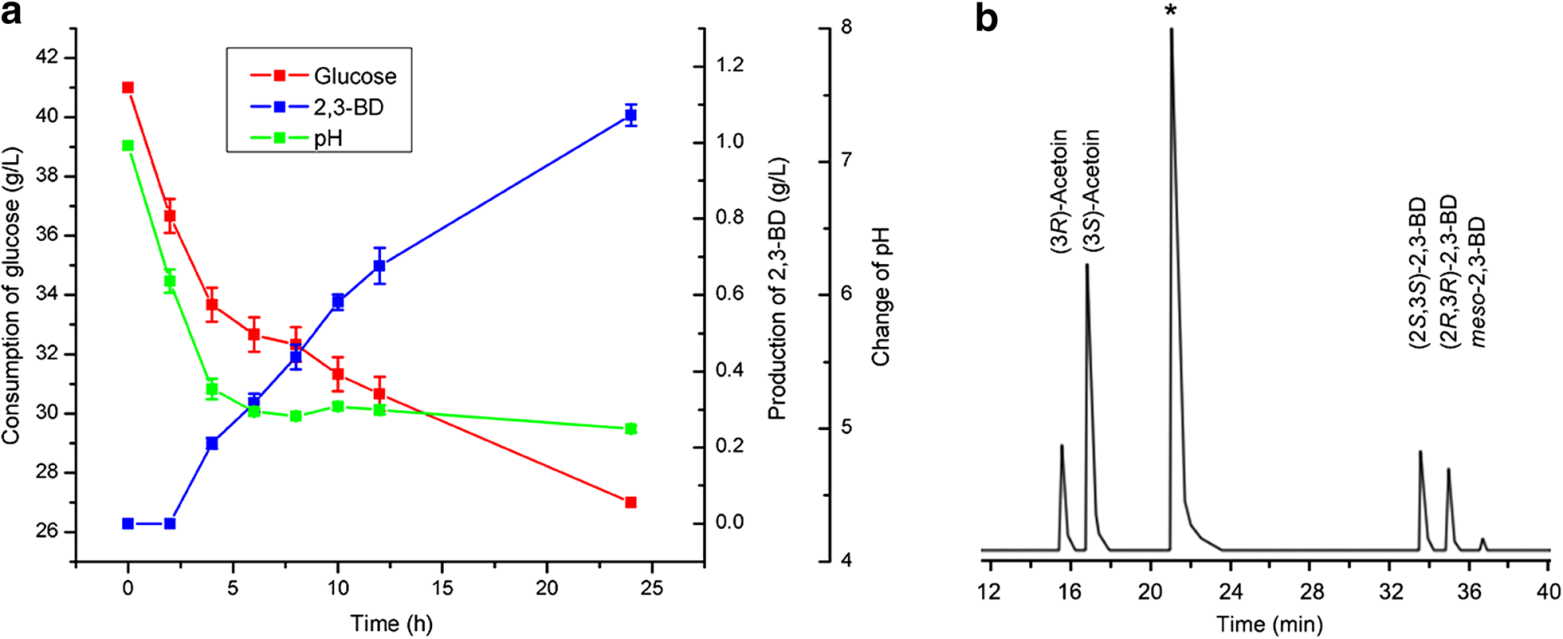
Production of 2,3-BD from glucose using whole cells of *E. coli* BL21 (pETDuet–P_T7_–*budB*–P_T7_–*budC*) without pH adjustment. **a** Time course of 2,3-BD production from glucose using whole cells of *E. coli* BL21 (pETDuet–P_T7_–*budB*–P_T7_–*budC*) without pH adjustment. **b** Chromatograph profile of 2,3-BD produced from glucose using whole cells of *E. coli* BL21 (pETDuet–P_T7_–*budB*–P_T7_–*budC*) without pH adjustment (*asterisk* isoamyl alcohol was used as the internal standard)

The stereoisomeric composition of 2,3-BD produced by *E. coli* BL21 (pETDuet–P_T7_–*budB*–P_T7_–*budC*) was analyzed with GC. As shown in Fig. 3b, a mixture of 2,3-BD was obtained, which contained only 57.4 % of (2*S*,3*S*)-2,3-BD. This result is rather inconsistent with our expectation. Thus, we analyzed the bioconversion system and drew the time course of the process. As shown in Fig. 3a, during the bioconversion process, pH was decreased from 7.0 to 5.0 due to production of organic acids. Thus, we expected that the decrease in pH might be the reason of the low stereoisomeric purity of the (2*S*,3*S*)-2,3-BD.

**Fig. 4:**
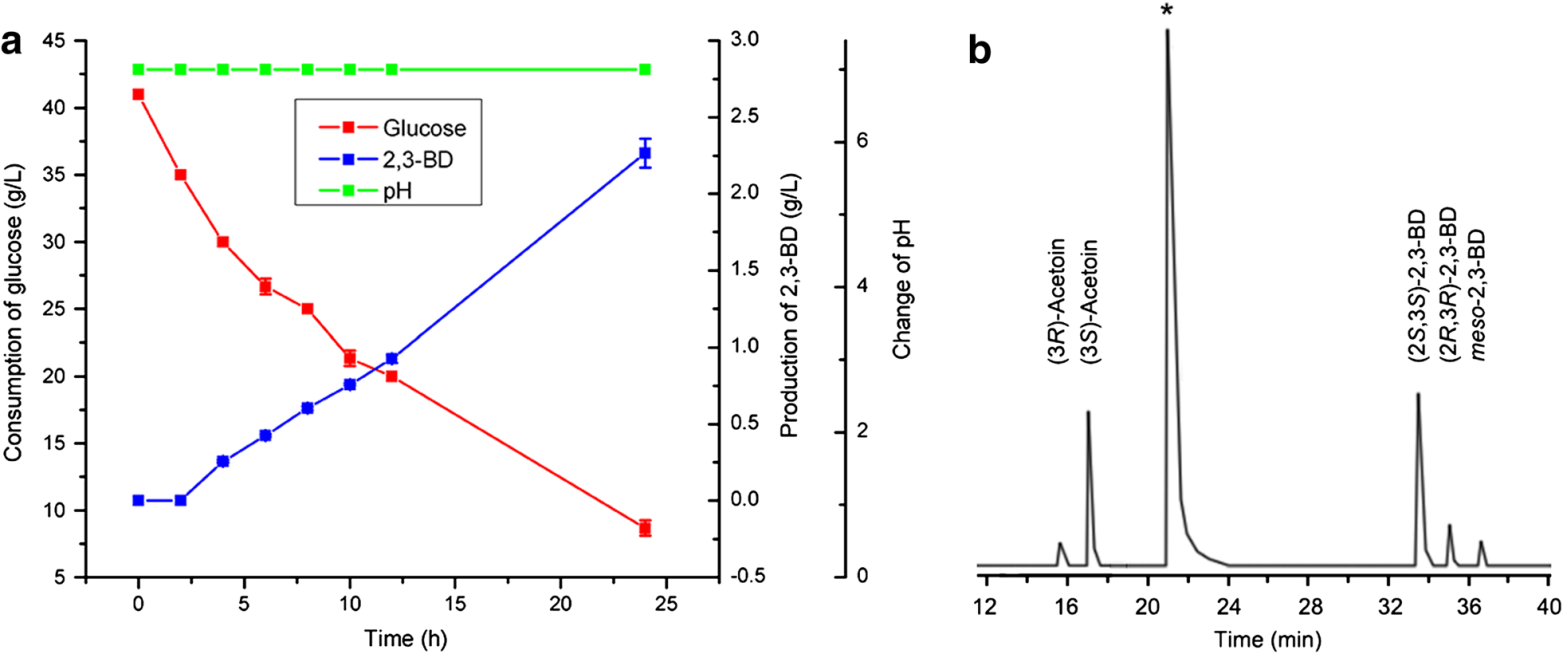
Production of 2,3-BD from glucose using whole cells of *E. coli* BL21 (pETDuet–P_T7_–*budB*–P_T7_–*budC*) with pH adjustment. **a** Time course of 2,3-BD production from glucose using whole cells of *E. coli* BL21 (pETDuet–P_T7_–*budB*–P_T7_–*budC*) with pH adjustment. **b** Chromatograph profile of 2,3-BD produced from glucose using whole cells of *E. coli* BL21 (pETDuet–P_T7_–*budB*–P_T7_–*budC*) with pH adjustment (*asterisk* isoamyl alcohol was used as the internal standard)

Then, during the bioconversion process, 10 M NaOH was added periodically to control the pH of the bioconversion system at 7.0 (Fig. 4a). As shown in Fig. 4b, pH adjustment resulted in a higher (2*S*,3*S*)-2,3-BD concentration, yield and especially a higher stereoisomeric purity of (2*S*,3*S*)-2,3-BD. Consequently, pH was maintained at 7.0 during subsequent bioconversions.

### Effect of temperature on production of (2*S*,3*S*)-2,3-BD by recombinant *E. coli*

**Fig. 5:**
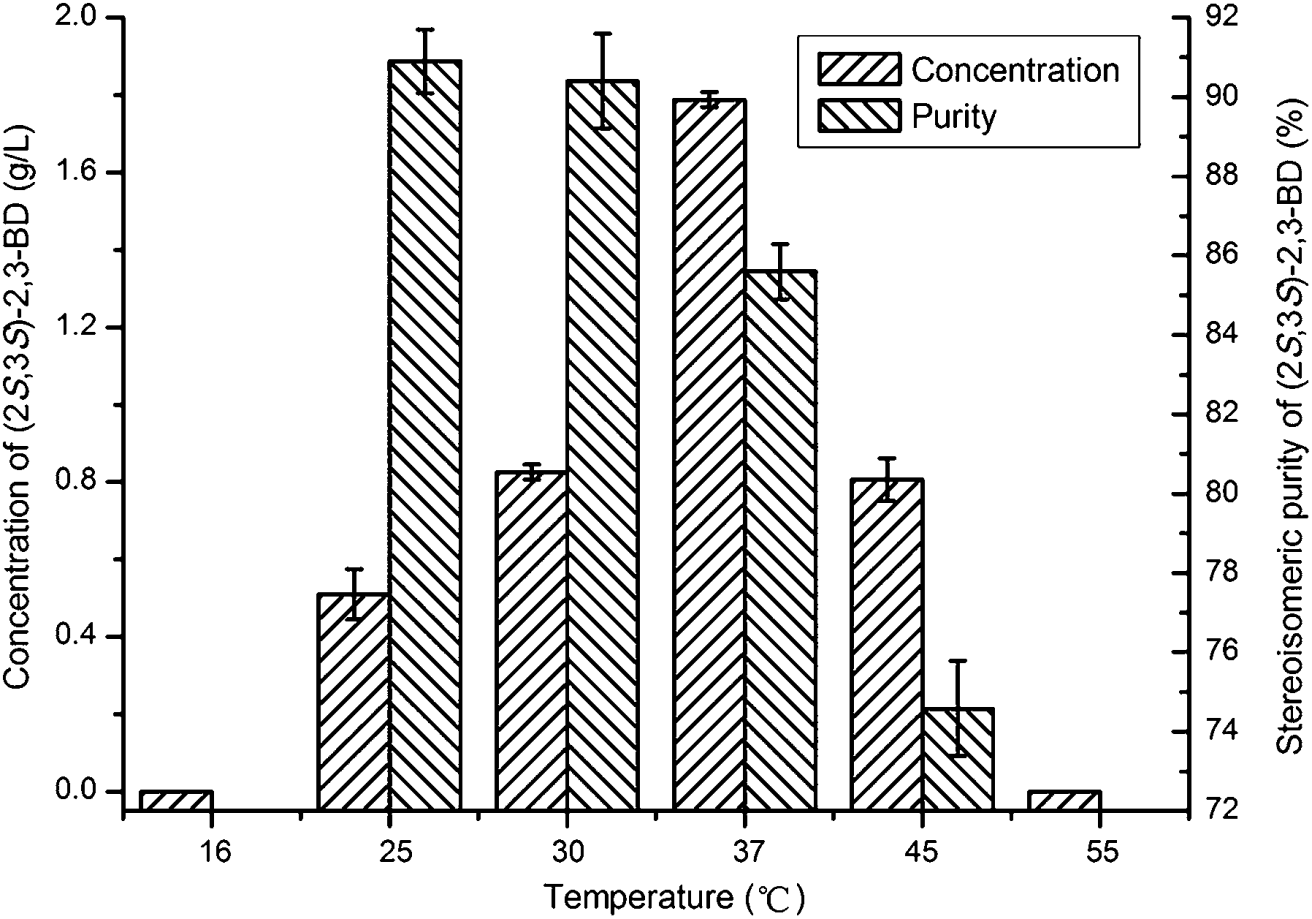
Effect of temperature on (2*S*,3*S*)-2,3-BD production by recombinant *E. coli* BL21 (pETDuet–P_T7_–*budB*–P_T7_–*budC*)

Efficiency of the bioconversion processes and non-enzymatic reaction is temperature dependent. Thus, in this study, the effects of temperature (16, 25, 30, 37, 45 and 55 °C) on (2*S*,3*S*)-2,3-BD production were also examined. As shown in Fig. 5, the highest (2*S*,3*S*)-2,3-BD concentration was obtained when the temperature was maintained at 37 °C. However, the stereoisomeric purity of (2*S*,3*S*)-2,3-BD was much lower than that of 25 and 30 °C. The stereoisomeric purity of (2*S*,3*S*)-2,3-BD is rather important for its utilization as the building block in asymmetric synthesis. Since both high product concentration and stereoisomeric purity could be obtained at 30 °C, this temperature was chosen for subsequent bioconversions.

### Effect of Fe^3+^ addition on (2*S*,3*S*)-2,3-BD production

To achieve higher (2*S*,3*S*)-2,3-BD concentration, non-enzymatic oxidative decarboxylation of α-acetolactate should be enhanced. It was reported that conversion of α-acetolactate into diacetyl could be enhanced by addition of Fe^3+^ [35]. To study the effect of the addition of Fe^3+^ on (2*S*,3*S*)-2,3-BD production, 10 mM FeCl_3_ or 10 mM ethylenediaminetetraacetic acid ferric sodium salt (EDTA-FeNa) was added at the beginning of the bioconversion process. The (2*S*,3*S*)-2,3-BD production and glucose consumption were detected after 24 h bioconversion. As shown in Table 3, addition of FeCl_3_ would result in a higher (2*S*,3*S*)-2,3-BD yield.

**Table 3:** Effects of Fe^3+^ addition on glucose consumption, 2,3-BD production, yield and purity of (2*S*,3*S*)-2,3-BD

Condition	Glucose consumed (g/L)	2,3-BD (g/L)	2,3-BD yield (g/g)	Purity of (2*S*,3*S*)-2,3-BD
Blank	32.00 ± 1.00	1.93 ± 0.04	0.060 ± 0.001	0.84 ± 0.02
10 mM FeCl_3_	27.00 ± 1.00	1.73 ± 0.03	0.064 ± 0.001	0.86 ± 0.02
10 mM EDTA-FeNa	24.67 ± 1.53	0.41 ± 0.03	0.017 ± 0.001	1.00 ± 0.00

Data are the mean ± standard deviations (SDs) from three parallel experiments

### Batch bioconversion under optimal conditions

Combining the results mentioned above, an optimal system for the production of (2*S*,3*S*)-2,3-BD from glucose was developed. Bioconversion was conducted at 30 °C in 50-mL shake flasks containing 20 mL medium. The medium consisted of 40 g/L glucose, 10 mM FeCl_3_ and 5 g DCW/L whole cells of *E. coli* BL21 (pETDuet–P_T7_–*budB*–P_T7_–*budC*). The pH was maintained at 7.0 through periodical addition of 10 M NaOH.

**Fig. 6:**
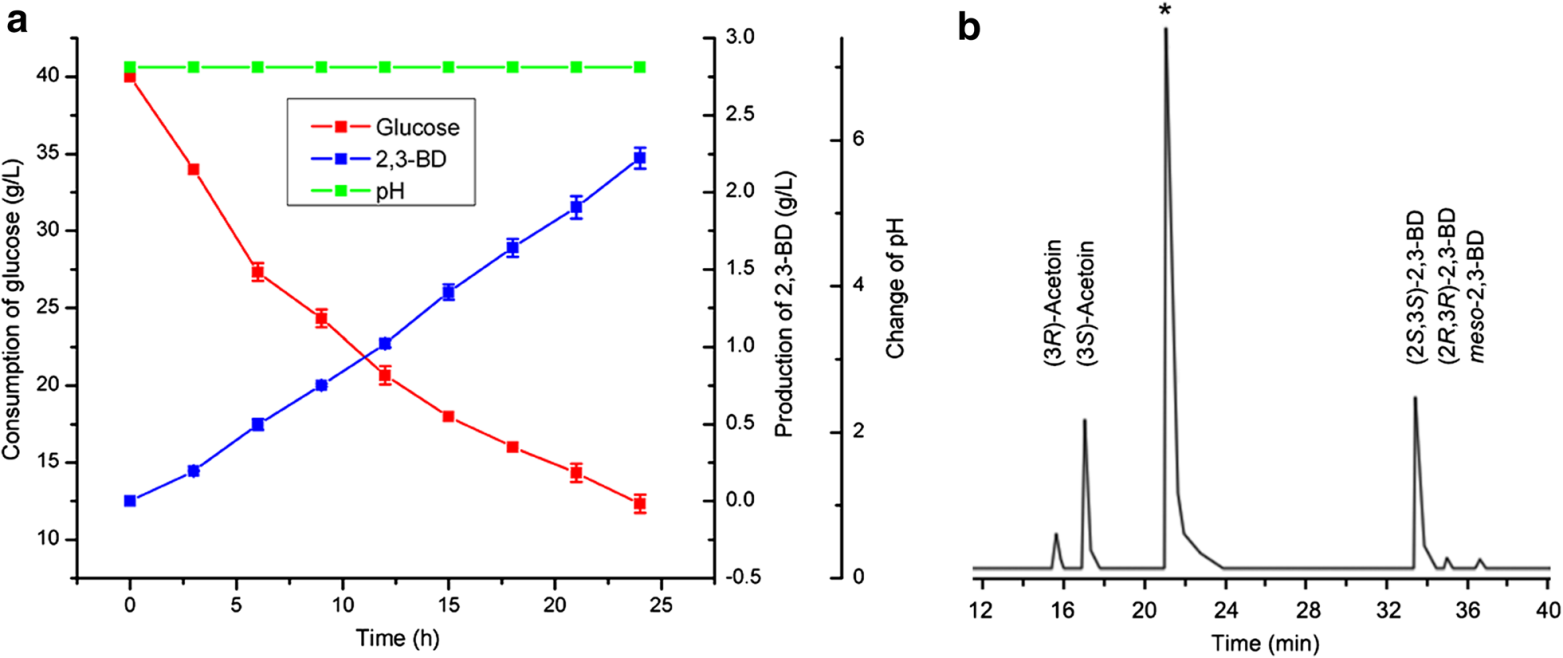
Production of 2,3-BD from glucose using whole cells of *E. coli* BL21 (pETDuet–P_T7_–*budB*–P_T7_–*budC*) with Fe^3+^ addition. **a** Time course of 2,3-BD production from glucose using whole cells of *E. coli* BL21 (pETDuet–P_T7_–*budB*–P_T7_–*budC*) with Fe^3+^ addition. **b** Chromatograph profile of 2,3-BD produced from glucose using whole cells of *E. coli* BL21 (pETDuet–P_T7_–*budB*–P_T7_–*budC*) with Fe^3+^ addition (*asterisk* isoamyl alcohol was used as the internal standard)

As shown in Fig. 6, 2.2 g/L (2*S*,3*S*)-2,3-BD was obtained from 26.7 g/L glucose after 24 h of bioconversion. The yield of (2*S*,3*S*)-2,3-BD was at 16.1 % of the theoretical value (Fig. 6a). The stereoisomeric purity of the (2*S*,3*S*)-2,3-BD produced by strain *E. coli* BL21 (pETDuet–P_T7_–*budB*–P_T7_–*budC*) was 95 % (Fig. 6b). Just like the situation of the fermentation process, the (2*S*,3*S*)-2,3-BD was produced through a mixed acid pathway during the bioconversion process. By-products including acetate, lactate, formate, succinate, acetoin and ethanol were detected in the bioconversion system (Additional file 1: Figure S2). Further enhancement of (2*S*,3*S*)-2,3-BD production might be acquired through elimination of these by-products through gene knockout.

Several biotechnological routes including enzymatic or whole-cell conversion methods have been used to produce (2*S*,3*S*)-2,3-BD (Table 4). Among all of the reported biotechnological processes, Wang et al. obtained the highest (2*S*,3*S*)-2,3-BD concentration of 31.7 g/L from diacetyl using recombinant *E. coli* coexpressing FDH and *meso*-BDH [23]. Efforts have also been tried to increase (2*S*,3*S*)-2,3-BD production through biotransformation of racemic acetoin [25, 26]. However, the prices of these two substrates, acetoin and diacetyl, are too high for the actual production of (2*S*,3*S*)-2,3-BD. Using racemic 2,3-BD as the substrate, the recombinant *E. coli* strain that coexpressed (2*R*,3*R*)-2,3-BDH and NADH oxidase produced (2*S*,3*S*)-2,3-BD at a concentration of 2.4 g/L [28]. Due to the low content of (2*S*,3*S*)-2,3-BD in the mixture of 2,3-BD (often lower than 10 %), the yield of (2*S*,3*S*)-2,3-BD was rather low, which increased the real substrate cost of the biocatalystic process.

**Table 4:** Researches on the production of (2*S*,3*S*)-2,3-BD using varying substrates

Biocatalyst	Substrate	(2*S*,3*S*)-2,3-BD (g/L)	Yield (g/g)	Co-substrate	Cost ($/kg)	References
*E. coli* coexpressing formate dehydrogenase from *Candida boidinii* NCYC 1513 and *meso*-BDH from *E. cloacae* subsp. *dissolvens* SDM	Diacetyl	31.7	0.90	Formate	24.4	[23]
*E. coli* expressing *meso*-BDH from *E. cloacae* subsp. *dissolvens* SDM	Diacetyl	26.8	0.67	Glucose	32.8	[24]
*E. coli* JM109 coexpressing *meso*-BDH from *K. pneumoniae* and (2*S*,3*S*)-2,3-BDH from *Brevibacterium saccharolyticum* C-1012	Diacetyl	2.2	0.93	Glucose	23.7	[25]
*E. coli* JM109 expressing (2*S*,3*S*)-2,3-BDH from *Br. saccharolyticum*	Racemic acetoin	3.7	0.37	Glucose	85.7	[26]
*E. coli* expressing acetoin reductase from *Rhodococcus erythropolis* WZ010	Diacetyl	5.3	1.03	NADH	21.4	[27]
*E. coli* BL21 coexpressing (2*R*,3*R*)-2,3-BDH from *Bacillus subtilis* 168 and NADH oxidase from *Lactobacillus brevis* CICC 6004	2,3-BD	2.4	0.12		13.3	[28]
*E. coli* BL21 (DE3) coexpressing ALS and *meso*-BDH from *E. cloacae* subsp. *dissolvens* SDM	Glucose	2.2	0.08		9.4	This work

Cost analyses of substrates were performed as described in Additional file 1

It has been reported that the cost of substrates accounted for more than about 30 % of total cost of 2,3-BD production. In this work, we constructed a recombinant *E. coli* coexpressing ALS and *meso*-BDH for the production of (2*S*,3*S*)-2,3-BD from a cheap substrate, glucose. The product concentration of our system was lower than that of processes using other substrates (Table 4). However, the cost analyses indicated that our process could produce (2*S*,3*S*)-2,3-BD at a rather low substrate cost (Additional file 1: Table S1). Thus, the method presented in this work would be a promising process for (2*S*,3*S*)-2,3-BD production.

## Conclusions

An efficient process for (2*S*,3*S*)-2,3-BD production from glucose was developed by using recombinant *E. coli* coexpressing ALS and *meso*-BDH. Under optimal conditions, the bioconversion process could produce 2.2 g/L (2*S*,3*S*)-2,3-BD with a yield of 0.08 g/g glucose. The stereoisomeric purity of (2*S*,3*S*)-2,3-BD produced from glucose was 95 %. The cost analysis suggests that the novel bioconversion system can serve as a promising choice for the industrial production of (2*S*,3*S*)-2,3-BD.

## Methods

### Materials

(2*S*,3*S*)-2,3-BD (99.0 %), (2*R*,3*R*)-2,3-BD (98.0 %) and *meso*-2,3-BD (98.0 %) were purchased from ACROS (The Kingdom of Belgium). Racemic acetoin (99.0 %) was purchased from Apple Flavor & Fragrance Group Co. (Shanghai, China). Diacetyl, ampicillin, isopropyl-β-d-thiogalactoside (IPTG), reduced nicotinamide adenine dinucleotide (NADH) and nicotinamide adenine dinucleotide (NAD) were purchased from Sigma. PCR primers were prepared by Sangon (Shanghai, China). Fast Pfu DNA polymerase was purchased from TransGen Biotech (Beijing, China). T_4_ DNA ligase and restriction endonucleases were obtained from Fermentas (Lithuania). All other chemicals were of analytical grade and commercially available.

### Bacterial strains and plasmids

The bacterial strains and plasmids used in this study are listed in Table 5. *E. coli* DH5α and BL21 (DE3) were used as cloning and expression host, respectively. The pEASY-Blunt cloning vector (TransGen Biotech, China) was used for gene cloning, pETDuet-1 and pET28a were used for gene expression. *E. cloacae* strain SDM was cultured in a medium containing the following (g/L) at pH 7.0: glucose, 15; peptone, 10; yeast extract, 5; KCl, 5. Luria–Bertani (LB) medium was used for *E. coli* cultivations. Ampicillin was used at a concentration of 100 μg/mL and kanamycin was used at a concentration of 50 μg/mL.

**Table 5:** Bacterial strains, plasmids and primers used in this study

Name	Characteristic	References
Strains		
*E. coli* DH5α	F^−^ φ80 lacZΔM15 Δ(lacZYA-argF)U169 recA1 endA1 hsdR17(rk^−^, mk^+^) supE44λ-thi-1	Novagen
*E. coli* BL21(DE3)	F^−^ ompT gal dcm lon hsdS_B_(r_B_^−^m_B_^−^) λ(DE3)	Novagen
*E. cloacae* subsp. *dissolvens* SDM	Wild type	[5]
*E. coli* BL21 (pET28a–*lysR*–P_abc_–*budB*–*budC*)	*E. coli* BL21(DE3) harboring pET28a–*lysR*–P_abc_–*budB*–*budC*	This study
*E. coli* BL21 (pETDuet–P_T7_–*budB*–P_T7_–*budC*)	*E. coli* BL21(DE3) harboring pETDuet–P_T7_–*budB*–P_T7_–*budC*	This study
Plasmids		
pET28a	Expression vector, Kan^r^	Novagen
pETDute-1	Expression vector, Amp^r^	Novagen
pEasy-Blunt	Kan^r^ Amp^r^ oripUC	Transgen
pET28a–*lysR*–P_abc_–*budB*–*budC*	*lysR*, P_abc_, *budB* and *budC* of *E. cloacae* subsp. *dissolvens* SDM were ligated and cloned into the multiple clone site of pET28a	This study
pETDuet–P_T7_–*budB*–P_T7_–*budC*	*budB* and *budC* from *Enterobacter cloacae* subsp. *dissolvens* SDM were cloned into the two multiple clone sites of pETDuet-1	This study
Primers		
*budB*-F (*Bgl*II)	5′-AGATCTAGTGAACAGTGATAAACAG-3′	This study
*budB*-R (*Xho*I)	5′-CTCGAGTCACAAAATCTGGCTGAGA-3′	This study
*budC*-F (*EcoR*I)	5′-GAATTCAATGCAAAAAGTTGCTCTCG-3′	This study
*budC*-R (*Hin*dIII)	5′-AAGCTTTTAATTGAATACCATCCCACCGT-3′	This study
*lysR*–*P*_*abc*_-F (*Bgl*II)	5′-CGGTAGATCTCTACTCCTCGCTTATCATCG-3′	This study
*lysR*–*P*_*abc*_-R	5′-CTCACTGTTCATGCTCGTCCTCTTC-3′	This study
*budB*–*budC*-F	5′-GAAGAGGACGAGCATGAACAGTGAG-3′	This study
*budB*–*budC*-R (*Hin*dIII)	5′-GCCTAAGCTTTTAGTTGAACACCATCCCA-3′	This study

### Construction of plasmid pETDuet–P_T7_**–***budB***–**P_T7_**–***budC*

*E. cloacae* strain SDM genomic DNAs were extracted with the Wizard Genomic DNA Purification Kit (Promega, Madison, WI, USA). The *budB* gene was amplified by PCR using forward primer *budB*-F with a *Bgl*II restriction site insertion and reverse primer *budB*-R with a *Xho*I restriction site insertion (Table 5). The PCR product was firstly ligated to the pEASY-Blunt vector, and the resulting plasmid was designated pEASY-Blunt–*budB*. Next, pEASY-Blunt–*budB* was digested with *Bgl*II and *Xho*I, and the gel-purified *budB* fragment was ligated to the pETDuet-1 vector digested with the same restriction enzymes. The resulting plasmid was designated pETDuet–P_T7_–*budB*. Using the same process as described above, the *budC* gene fragment was obtained from the genome of *E. cloacae* strain SDM using primers *budC*-F (with the *Eco*RI restriction site) and *budC*-R (with the *Hin*dIII restriction site) (Table 5), and the pETDuet–P_T7_–*budB*–P_T7_–*budC* was constructed based on the pETDuet–P_T7_–*budB*.

### Construction of plasmid pET28a–*lysR*–P_abc_–*budB*–*budC*

The *lysR*, *P*_*abc*_, *budB* and *budC* are sequentially clustered in one operon in the *E. cloacae* SDM. The fragments *lysR*–P_abc_ and *budB*–*budC* of *E. cloacae* SDM were amplified through PCR with the primer pairs *lysR*–P_abc_-F(*Bgl*II)/*lysR*–P_abc_-R(overlap) and *budB*–*budC*-F(overlap)/*budB*–*budC*-R(*Hin*dIII), respectively. Then the fragments *lysR*–P_abc_ and *budB*–*budC* of *E. cloacae* SDM were then ligated through gene splicing by overlap extension using the primers *lysR*–P_abc_-F(*Bgl*II) and *budB*–*budC*-R(*Hin*dIII). Then fragment *lysR*–P_abc_–*budB*–*budC* was ligated to the pEASY-Blunt vector to get pEASY-Blunt–*lysR*–P_abc_–*budB*–*budC*. Then, pEASY-Blunt–*lysR*–P_abc_–*budB*–*budC* was digested with *Bgl*II and *Hin*dIII, and the gel-purified *lysR*–P_abc_–*budB*–*budC* fragment was ligated to the pET28a vector digested with the same restriction enzymes. The resulting plasmid was designated pET28a–*lysR*–P_abc_–*budB*–*budC*.

### Biocatalyst preparation and bioconversion conditions

LB medium supplemented with 100 μg/mL ampicillin or 50 μg/mL kanamycin was used to cultivate *E. coli* BL21 (pETDuet-1) and *E. coli* BL21 (pET28a–*lysR*–P_abc_–*budB*–*budC*). The cells of the strains were grown for 12 h, then centrifuged at 13,000×*g* for 5 min, and washed twice with 67 mM phosphate buffer (pH 7.4). The *E. coli* BL21 (pETDuet–P_T7_–*budB*–P_T7_–budC) were grown in LB medium containing 100 μg/mL of ampicillin at 37 °C on a rotary shaker (180 rpm). The cultures were induced with 1 mM IPTG at an OD_620 nm_ of 0.6 at 16 °C for about 10 h. The cells were harvested by centrifugation at 6000×*g* for 5 min at 4 °C and then washed twice with 67 mM phosphate buffer (pH 7.4). The cell pellets were resuspended in 67 mM phosphate buffer (pH 7.4) as biocatalysts for further bioconversion study.

(2*S*,3*S*)-2,3-BD production was carried out using 40.0 g/L of glucose as the substrate and 5.0 g DCW/L whole cells of the recombinant as the biocatalysts. 20 mL of mixture with 10 mM Fe^3+^ was reacted at 30 °C and 180 rpm in 50 mL flasks. pH was controlled at 7.0 by adding 10 M NaOH.

### Enzyme activity assays

To measure enzyme activity, the cells of the different *E. coli* strains were resuspended in 67 mM phosphate buffer (pH 7.4) and disrupted with an ultrasonic cell-breaking apparatus (Xinzhi, Ningbo, China). Cell debris was removed through centrifugation at 13,000×*g* for 15 min. The resulting supernatants were used in the successive enzyme activity assays. All enzyme assays were performed at 30 °C with the proper enzyme in 67 mM phosphate buffer (pH 7.4).

Activity of ALS was measured by monitoring the conversion of pyruvate to α-acetolactate [36]. One unit of ALS activity was defined as the amount of enzyme that produced 1 μmol of α-acetolactate per minute.

ALDC activity was assayed by detecting the production of acetoin from α-acetolactate [37]. α-Acetolactate was prepared immediately before use of ethyl 2-acetoxy-2-methyl-acetoacetate, according to the protocol supplied by the manufacturer. One unit of ALDC activity was defined as the amount of protein that formed 1 μmol of acetoin per min.

*meso*-BDH activity was assayed by measuring the change in absorbance at 340 nm corresponding to the oxidation of NADH or reduction of NAD when diacetyl or acetoin was used as the substrate [38]. For the reduction reaction, 5 mM acetoin or diacetyl and 0.2 mM NADH were used for the enzyme assay, and 10 mM *meso*-2,3-BD and 1 mM NAD were used for the oxidation reactions. One unit of enzyme activity was defined as the amount of enzyme that consumed 1 μmol of NADH or produced 1 μmol of NADH per minute.

The protein concentration was determined by the Lowry procedure using bovine serum albumin as the standard [39].

### Analytical methods

Samples were withdrawn periodically and centrifuged at 12,000×*g* for 10 min. The concentration of glucose was measured enzymatically by a bio-analyzer (SBA-40D, Shandong Academy of Sciences, China) after diluting to an appropriate concentration. The concentrations of 2,3-BD were analyzed by GC (Varian 3800) as described previously [17]. The GC system was equipped with a capillary GC column (AT. SE-54, inside diameter, 0.32 mm; length, 30 m, Chromatographic Technology Center, Lanzhou Institute of Chemical Physics, China). The ratio of the three stereoisomers of 2,3-BD was analyzed by GC (Agilent GC6820) using a fused silica capillary column (Supelco Beta DEXTM 120, inside diameter 0.25 mm; length 30 m) [40]. The stereoisomeric purity of (2*S*,3*S*)-2,3-BD was defined as (([S])/([M] + [S] + [R])) × 100 %, where [S], [M] and [R] represent the concentrations of (2*S*,3*S*)-2,3-BD, *meso*-2,3-BD and (2*R*,3*R*)-2,3-BD, respectively.

## Additional file

**Additional file 1: Figure S1.** Verification of recombinant vectors.**Figure S2.** Time course of byproducts production from glucose using whole cells of* E. coli* BL21 (pETDuet-P_T7_-*bud* *B*-P_T7_-*bud* *C*) with Fe^3+^ addition.** Table S1.** Cost analyses of various (2*S*,3*S*)-2,3-BD production processes.

## Abbreviations

GC: gas chromatography
HPLC: high-performance liquid chromatography
2,3-BD: butane-2,3-diol
ALS: α-acetolactate synthase
ALDC: α-acetolactate decarboxylase
BDH: 2,3-BD dehydrogenase
NADH: reduced nicotinamide adenine dinucleotide
NAD: nicotinamide adenine dinucleotide
