# Supplementary material for: Metabolic engineering of Escherichia coli for production of (2S,3S)-butane-2,3-diol from glucose
Source: Biotechnol Biofuels. 2015 Sep 15;8:143. doi: 10.1186/s13068-015-0324-x (PMC4570510; doi:10.1186/s13068-015-0324-x)
Supplement: Supplementary file 1 — Additional file 1: Figure S1. Verification of recombinant vectors.Figure S2. Time course of byproducts production from glucose using whole cells of E. coli BL21 (pETDuet-PT7-bud B-PT7-bud C) with Fe3+ addition. Table S1. Cost analyses of various (2S,3S)-2,3-BD production processes. [file 13068_2015_324_MOESM1_ESM.doc]

**Metabolic engineering of *Escherichia coli* for production of (2*S*,3*S*)-butane-2,3-diol from glucose**

Haipei Chu^a^, Bo Xin^a^, Peihai Liu^c^,Yu Wang^b^, Lixiang Li^a^, Xiuxiu Liu^a^, Xuan Zhang ^a^, Cuiqing Ma^a^, Ping Xu^b^, and Chao Gao^a^*

^a^ State Key Laboratory of Microbial Technology, Shandong University, Jinan 250100, People’s Republic of China

^b^ State Key Laboratory of Microbial Metabolism, School of Life Sciences and Biotechnology, Shanghai Jiao Tong University, Shanghai 200240, People’s Republic of China

^c^ Rizhao Entry-Exit Inspection and Quarantine Bureau, Rizhao 276800, People’s Republic of China

 *Corresponding author

Authors emails:

HC: chuhp@163.com

BX: xinbosdu@gmail.com

HL: liuph_513@sina.com

YZ: wang_yu@sjtu.edu.cn

LL: [lilixiang@sdu.edu.cn](mailto:lilixiang@sdu.edu.cn)

XL: [1179796910@qq.com](mailto:1179796910@qq.com)

XZ: 809401655@qq.com

CM: macq@sdu.edu.cn

PX: [pingxu@sjtu.edu.cn](mailto:pingxu@sjtu.edu.cn)

CG: jieerbu@sdu.edu.cn

##
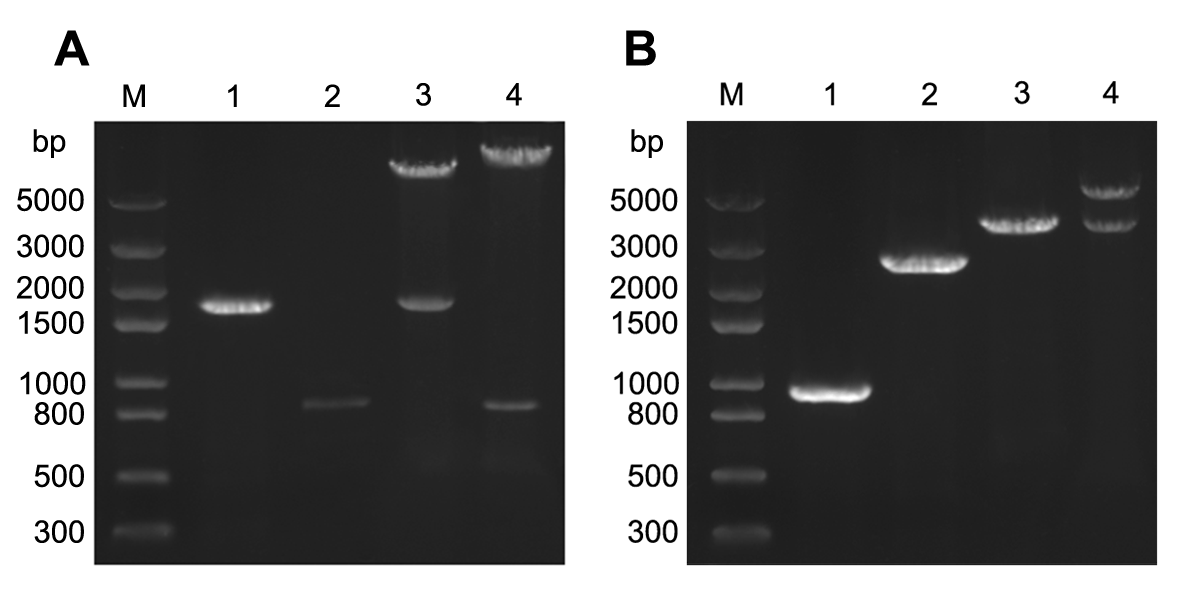


## Fig. S1 Verification of recombinant vectors.

## (A) Verification of pETDuet–P_T7_–*budB*–P_T7_–*budC.* Lane M, trans 5K marker; lane 1–2, PCR amplification of *budB* and *budC* from *E. cloacae* subsp. *dissolvens* SDM; lane 3, double enzyme digestion (*Bgl*II and *Xho*I) of pETDuet–P_T7_–*budB*–P_T7_–*budC*; lane 4, double enzyme digestion (*Eco*RI and *Hin*dIII) of pETDuet–P_T7_–*budB*–P_T7_–*budC*. (B) Verification of pET28a–*lysR*–P_abc_–*budB*–*budC.* Lane M, trans 5K marker; lane 1–2, PCR amplification of *lysR–*P_abc_ and *budB–budC* from *E. cloacae* subsp. *dissolvens* SDM; lane 3, gene splicing of *lysR–*P_abc_ and *budB–budC* from *E. cloacae* subsp. *dissolvens* SDM by overlap extension and gel extraction purification; lane 4, double enzyme digestion *(Bgl*II and *Hin*dIII) of pET28a–*lysR*–P_abc_–*budB*–*budC*.

#
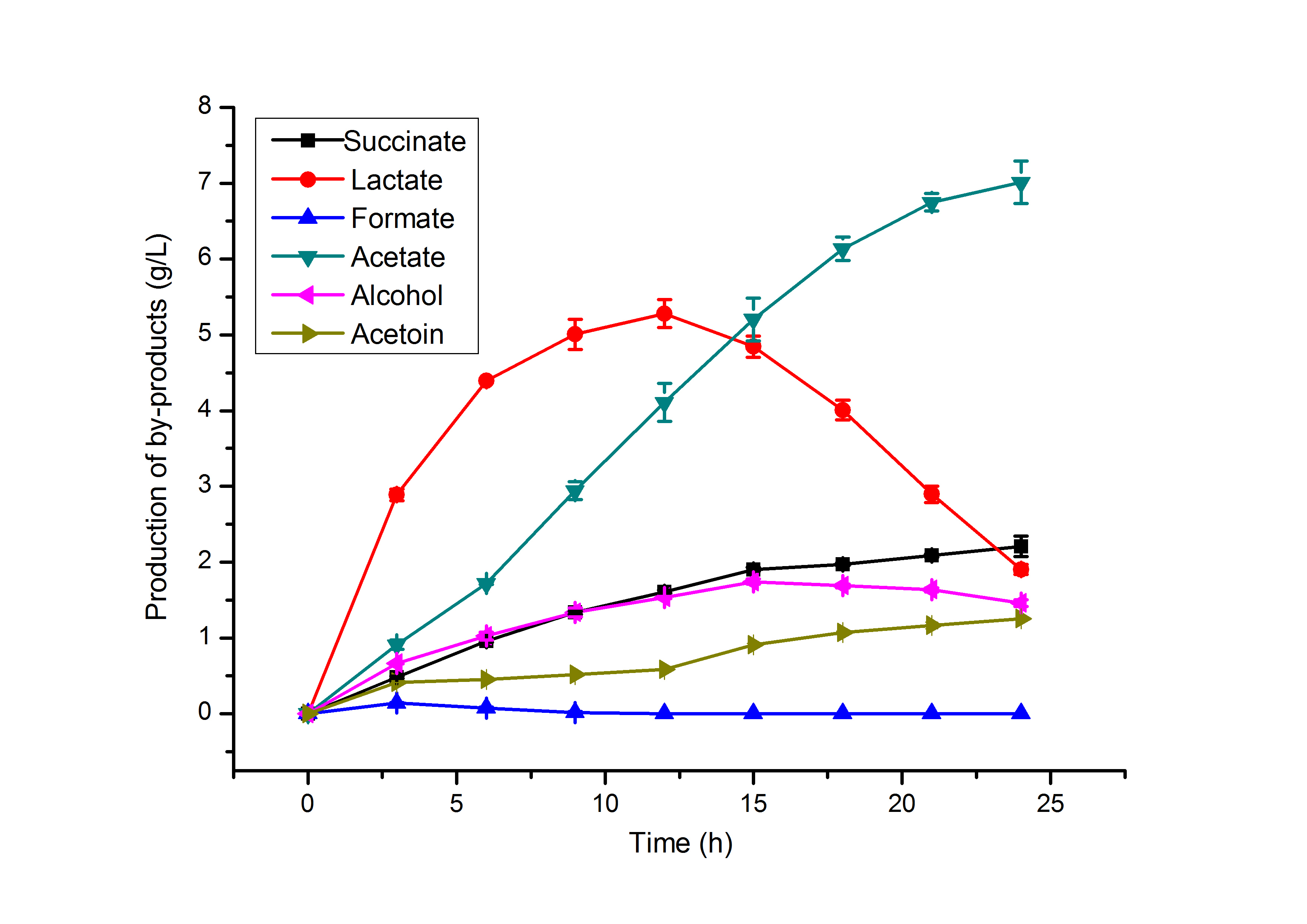


# Fig. S2 Time course of by-products production from glucose using whole cells of *E. coli* BL21 (pETDuet–P_T7_–*budB*–P_T7_–*budC*) with Fe^3+^ addition*.*

**Table S1 Cost analyses of various (2*S*,3*S*)-2,3-BD production processes**

| **Substrate** | **Price ($/kg)** | **(2*S*,3*S*)-2,3-BD yield (g/g)** | **Cost ($/kg (2*S*,3*S*)-2,3-BD)** | **Reference** |
| --- | --- | --- | --- | --- |
| Diacetyl | 22.0 | 0.90 | 24.4 | 1 |
| Diacetyl | 22.0 | 0.67 | 32.8 | 2 |
| Diacetyl | 22.0 | 0.93 | 23.7 | 3 |
| Racemic acetoin | 31.7 | 0.37 | 85.7 | 4 |
| Diacetyl | 22.0 | 1.03 | 21.4 | 5 |
| 2,3-BD | 1.6 | 0.12 | 13.3 | 6 |
| Glucose | 0.75 | 0.08 | 9.4 | This work |

The cost of (2*S*,3*S*)-2,3-BD was defined as , where P_substrate_ and Y_BD/substrare_ represent the price of substrates and yield of (2*S*,3*S*)-2,3-BD, respectively. The prices of glucose, diacetyl, acetoin and 2,3-BD were taken approximately as 0.80 $/kg^[7]^, 22.0 $/kg^[8]^, 31.7 $/kg^[9]^ and 1.6 $/kg^[10]^, respectively.

References

1. Wang Y, Li L, Ma C, Gao C, Tao F, Xu P (2013) Engineering of cofactor regeneration enhances (2*S*,3*S*)-2,3-butanediol production from diacetyl. Sci Rep 3:2643

2. Li L, Wang Y, Zhang L, Ma C, Wang A, Tao F, et al (2012) Biocatalytic production of (2*S*,3*S*)-2,3-butanediol from diacetyl using whole cells of engineered *Escherichia coli*. Bioresour Technol 115:111–116

3. Ui S, Takusagawa Y, Sato T, Ohtsuki T, Mimura A, Ohkuma M, et al (2004) Production of l-2,3-butanediol by a new pathway constructed in *Escherichia coli*. Lett Appl Microbiol 39:533–537

4. Ui S, Takusagawa Y, Ohtsuki T, Mimura A, Ohkuma M, Kudo T (2001) Stereochemical applications of the expression of the l-2,3-butanediol dehydrogenase gene in *Escherichia coli*. Lett Appl Microbiol 32:93–98

5. Wang Z, Song Q, Yu M, Wang Y, Xiong B, Zhang Y, et al (2014) Characterization of a stereospecific acetoin (diacetyl) reductase from *Rhodococcus erythropolis* WZ010 and its application for the synthesis of (2*S*,3*S*)-2,3-butanediol. Appl Microbiol Biotechnol 98:641–650

6. Xiao Z, Lv C, Gao C, Qin J, Ma C, Liu Z, et al (2010) A novel whole-cell biocatalyst with NAD^+^ regeneration for production of chiral chemicals. PLoS One 5:e8860

7. World and U.S. Sugar and Corn Sweetener Prices (2015) US Department of Agriculture. Sugar and sweeteners yearbook tables. Table 7 <http://www.ers.usda.gov/data-products/sugar-and-sweeteners-yearbook-tables.aspx>

8. Eiteman MA, Altman E, Zhu Y (2014) Microbial production of pyruvate and other metabolites. US patent 8652825

9. Xiao Z, Lu JR (2014) Strategies for enhancing fermentative production of acetoin: A review. Biotechnol Adv 32:492–503

10. Shrivastav A, Lee J, Kim HY, Kim YR (2013) Recent insights in the removal of *Klebseilla* pathogenicity factors for the industrial production of 2,3-butanediol. J Microbiol Biotechnol 23:885–896
